# Supplementary material for: Maternal Pregnancy and Pre-Pregnancy Weight and Behavioural Outcomes in Children
Source: Behav Sci (Basel). 2024 Jan 12;14(1):49. doi: 10.3390/bs14010049 (PMC10812996; doi:10.3390/bs14010049)
Supplement: Supplementary file 1 [file behavsci-14-00049-s001.zip › behavsci-2756067-supplementary.pdf]

Table S1. Characteristics of mothers and children with data on SDQ compared with those with no SDQ data.

**Key variables**	**At 3 years, n (%)**		*p*-value*	**At 16 years, n (%)**		*p*-value*
	Completed data on SDQ, n = 8,317 (78.7)	Missed/lost follow-up, *n* = 2,254) (21.3)		Completed data on SDQ (*n* = 4,607)	Missed/lost follow-up (*n* = 5,964)	
Child's sex			0.238			< 0.001
Male	4,264 (51.3)	1,124 (49.9)		2,218 (48.1)	3,170 (53.2)	
Female	4,053 (48.7)	1,130 (50.1)		2,389 (51.9)	2,794 (46.8)	
Maternal education#			< 0.001			< 0.001
Certificate of secondary education	939 (12.0)	381 (2.0)		326 (7.4)	994 (1.5)	
Vocational	738 (9.5)	223 (12.9)		322 (7.3)	639 (12.5)	
O level	2,907 (37.2)	623 (36.0)		1,572 (35.5)	1,958 (38.3)	
A level	2,004 (25.7)	343 (19.8)		1,297 (29.3)	1,050 (20.5)	
Degree	1,221 (15.6)	160 (9.3)		911 (20.6)	470 (9.2)	
Marital status			< 0.001			< 0.001
Married	6,588 (80.3)	1,389 (65.8)		3,820 (83.6)	4,157 (72.3)	
Never married	1,199 (14.6)	560 (26.5)		542 (11.9)	1,217 (21.2)	
Widowed/divorced/Separated	422 (5.1)	1,63 (7.7)		209 (4.6)	376 (6.5)	
Parity			0.003			< 0.001
Nullipara	3,649 (44.9)	840 (41.2)		2,168 (47.9)	2,321 (41.2)	
Multipara	4,476 (55.1)	1,198 (58.8)		2,362 (52.1)	3,312 (58.8)	
Alcohol drinking in pregnancy			0.023			0.73
Yes	1,252 (15.3)	358 (17.3))		709 (15.5)	901 (15.8)	
No	6,954 (84.7)	1,713 (82.7)		3,857 (84.5)	4,810 (84.2)	
Smoking during pregnancy			< 0.001			< 0.001
Yes	1,691 (20.5)	683 (32.6)		689 (15.0)	1,685 (29.3)	
No	6,549 (79.5)	1,414 (67.4)		3,895 (85.0)	4,068 (70.7)	

Table S1. Continued.

Maternal antenatal anxiety symptoms			< 0.001			< 0.001
Yes	1,582 (20.3)	540 (30.0)		801 (18.4)	1,321 (25.2)	
No	6,209 (79.7)	1,260 (70.0)		3,550 (81.6)	3,919 (74.9)	
Maternal antenatal depressive symptoms			< 0.001			< 0.001
Yes	1,348 (17.0)	463 (24.7)		658 (14.8)	1,153 (21.4)	
No	6,604 (83.0)	1,412 (75.3)		3,779 (85.2)	4,237 (78.6)	
Pre-pregnancy BMI category (Kg/m2)			0.001			< 0.001
<18.5	372 (4.5)	520 (6.6)		186 (4.0)	334 (5.6)	
18.5 – 24.99	6,225 (74.8)	7,855 (72.3)		3,566 (77.4)	4,289 (71.9)	
25 – 29.99	1,261 (15.2)	1,607 (15.3)		649 (14.1)	958 (16.1)	
≥30	459 (5.5)	589 (5.8)		206 (4.5)	383 (6.4)	
Gestational weight gain, IOM category			0.063			0.074
Inadequate (less than recommended)	2,606 (32.7)	749 (35.2)		1,407 (33.4)	1,769 (33.4)	
Adequate (recommended)	3,158 (39.7)	793 (37.3)		1,694 (40.2)	2,030 (38.3)	
Excessive (more than recommended)	2,198 (27.6)	586 (25.7)		1,114 (26.4)	1,501 (28.3)	

**p*-values correspond to Pearson's chi-square test.

#O level indicates examinations taken and passed at 16 years of age. A level: examinations taken and passed at 18 years of age on leaving secondary school.

Table S2. Summary statistics of SDQ scores

Behavioural outcomes	Mean (SD)				
	3 years	7 years	9 years	11 years	16 years
Total difficulties	12.5 (5.65)	7.45 (4.76)	6.84 (4.94)	6.53 (4..95)	6.17 (4.76)
Hyper activity	2.62 (1.81)	3.37 (2.36)	2.94 (2.25)	2.77 (2.23)	2.55 (2.11)
Conduct problems	3.54 (2.21)	1.61 (1.47)	1.29 (1.44)	1.21 (1.43)	1.03 (1.35)
Emotional symptoms	2.55 (1.73)	1.51 (1.67)	1.52 (1.76)	1.47 (1.73)	1.50 (1.86)
Peer problems	---	1.05 (1.42)	1.12 (1.51)	1.11 (1.55)	1.11 (1.50)
Prosocial behaviour	---	8.16 (1.76)	8.32 (1.66)	8.33 (1.68)	8.0 (1.87)

SD = Standard deviation. "---" Not measured/no data available

Total difficulties score range: 0–40; other domains score range: 0–10. Higher scores represent higher problems except for pro-social behaviour, in which lower scores represent greater difficulties.

Table S3. Association between pre-pregnancy BMI and behavioural problems in children over time using continuous SDQ scores.

Pre-pregnancy BMI (Kg/m^2) and gestational weight gain	Unadjusted β (95% CI)					
	Total behavioural difficulties	Hyperactivity/ inattention problems	Conduct problems	Emotional symptoms	Peer-relationship problems	Pro-social behaviours
Pre-pregnancy BMI						
<18.5	0.96 (0.48, 1.42)	0.10 (-0.08, 0.28)	0.19 (0.05, 0.32)	0.22(0.08, 0.37)	0.09 (-0.06, 0.23)	-0.12 (-0.28, 0.05)
18.5 – 24.99	Reference	Reference	Reference	Reference	Reference	Reference
25 – 29.99	0.34 (0.08, 0.61)	0.08 (-0.03, 0.18)	0.10 (0.02, 0.18)	0.03 (-0.05, 0.11)	0.12 (0.04, 0.20)	0.04 (-0.05, 0.14)
≥30	0.45 (0.03, 0.88)	0.03 (-0.14, 0.19)	0.06 (-0.06, 0.18)	0.01 (-0.12, 0.14)	0.24 (0.12, 0.37)	0.25 (0.10, 0.40)
Gestational weight gain						
Inadequate	0.14 (-0.08, 0.36)	0.05 (-0.04, 0.14)	0.02 (-0.05, 0.08)	0.01 (-0.06, 0.76)	0.03 (-0.04, 0.09)	0.06 (-0.02, 0.14)
Adequate	Reference	Reference	Reference	Reference	Reference	Reference
Excessive	0.09 (-0.14, 0.33))	-0.07 (-0.17, 0.02)	-0.06 (-0.12, 0.01)	0.02 (-0.06, 0.09)	0.05 (-0.02, 0.12)	0.12 (0.04, 0.21)
	Adjusted β (95%CI)#					
Pre-pregnancy BMI						
<18.5	0.57 (0.12, 1.01)	0.10 (-0.08, 0.29)	0.19 (0.05, 0.32)	0.23 (0.09, 0.37)	0.09 (-0.05, 0.23)	-0.1 (-0.26, 0.07)
18.5 – 24.99	Reference	Reference	Reference	Reference	Reference	Reference
25 – 29.99	0.26 (0.02, 0.51)	0.08 (-0.03, 0.18)	0.10 (0.02, 0.17)	0.02 (-0.06, 0.10)	0.11 (0.03, 0.19)	0.04 (-0.06, 0.13)
≥30	0.23 (-0.18, 0.64)	0.03 (-0.14, 0.20)	0.06(-0.06, 0.18)	0.01 (-0.13, 0.13)	0.23 (0.10, 0.36)	0.22 (0.07, 0.38)
Gestational weight gain						
Inadequate	0.12 (-0.09, 0.34)	0.05 (-0.04, 0.14)	0.02 (-0.05, 0.08)	0.01 (-0.06, 0.08)	0.03 (-0.04, 0.10)	0.07 (-0.01, 0.14)
Adequate	Reference	Reference	Reference	Reference	Reference	Reference
Excessive	-0.11 (-0.34, 0.11)	-0.07 (-0.17, 0.02)	-0.06 (-0.13, 0.01)	0.01 (-0.06, 0.08)	0.04 (-0.03, 0.11)	0.11 (0.03, 0.19)

Adjusted for maternal age, education, marital status, parity, maternal smoking, alcohol use, anxiety and depressive symptoms during pregnancy and offspring sex.

Table S4. Association between pre-pregnancy BMI and behavioural problems in children over time using imputed data (GEE model).

Pre-pregnancy BMI (Kg/m²) and gestational weight gain	Adjusted OR (95%CI)					
	Total behavioural difficulties	Hyperactivity/ inattention problems	Conduct problems	Emotional symptoms	Peer-relationship problems	Pro-social behaviours
Pre-pregnancy BMI						
<18.5	1.08 (0.95-1.23)	1.08 (0.94-1.24)	1.00 (0.88-1.12)	1.20 (1.04-1.37)	0.99 (0.86-1.14)	1.24 (1.03-1.49)
18.5 – 24.99	1	1	1	1	1	1
25 – 29.99	1.04 (0.94-1.12)	1.02 (0.92-1.14)	1.08 (1.01-1.16)	0.99 (0.91-1.08)	1.13 (1.03-1.25)	0.86 (0.76-0.96)
≥30	1.01 (0.90-1.14)	0.98 (0.83-1.15)	1.10 (1.00-1.22)	1.02 (0.90-1.17)	1.25 (1.08-1.43)	0.65 (0.52-0.81)
Gestational weight gain						
Inadequate	1.01 (0.95-1.08)	1.05 (0.96-1.15)	0.98 (0.92-1.04)	1.01 (0.94-1.08)	1.02 (0.93-1.11)	0.93 (0.84-1.03)
Adequate	1	1	1	1	1	1
Excessive	0.96 (0.88-1.04)	0.99 (0.97-1.00)	0.93 (0.87-0.99)	1.02 (0.95-1.11)	1.08 (0.97-1.21)	0.78 (0.67-0.90)

Adjusted for maternal age, education, marital status, parity, maternal smoking, alcohol use, anxiety and depressive symptoms during pregnancy and offspring sex.

Table S5. Association between pre-pregnancy BMI and behavioural problems in children at each developmental periods (unadjusted [A] and adjusted [B] analysis).

Offspring age	Pre-pregnancy BMI (Kg/m²) and gestational weight gain	[A] Unadjusted; OR (95%CI)					
		Total behavioural difficulties	Emotional symptoms	Peer-relationship problems	Hyperactivity/ inattention problems	Conduct problems	Prosocial behaviours
3 years	Pre-Pregnancy BMI (*n* = 7142)						
	<18.5	1.32 (1.04-1.66)	1.36 (1.06-1.76)	…	0.92 (0.58-1.47)	1.23 (0.96-1.57)	…
	18.5 – 24.99	1	1	…	1	1	…
	25 – 29.99	1.07 (0.93-1.23)	1.09 (0.94-1.27)	…	1.12 (0.88-1.43)	1.19 (1.04-1.37)	…
	≥30	1.02 (0.82-1.27)	1.05 (0.83-1.34)	…	0.98 (0.65-1.49)	0.98 (0.79-1.22)	…
	Gestational weight gain (*n* = 6822)						
	Inadequate	1.08 (0.96-1.21)	1.04 (0.91-1.18)	…	1.12 (0.90-1.39)	1.05 (0.93-1.18)	…
	Adequate	1	1	…	1	1	…
	Excessive	1.00 (0.88-1.12)	1.16 (1.01-1.33)	…	1.04 (0.82-1.30)	0.89 (0.78-1.00)	…
7 years	Pre-Pregnancy BMI (*n* = 6159)						
	<18.5	1.66 (1.16-2.38)	1.85 (1.35-2.52)	1.51 (1.08-2.10)	1.32 (0.97-1.81)	1.13 (0.84-1.51)	1.20 (0.80-1.80)
	18.5 – 24.99	1	1	1	1	1	1
	25 – 29.99	1.18 (0.94-1.49)	0.94 (0.76-1.17)	1.19 (0.98-1.46)	1.13 (0.94-1.36)	1.07 (0.90-1.26)	1.02 (0.80-1.30)
	≥30	1.26 (0.88-1.81)	0.94 (0.66-1.33)	1.35 (0.99-1.84)	1.21 (0.91-1.62)	1.22 (0.94-1.59)	0.76 (0.49-1.18)
	Gestational weight gain (*n* = 5912)						
	Inadequate	1.12 (0.92-1.37)	0.98 (0.81-1.17)	0.90 (0.75-1.08)	1.04 (0.88-1.21)	1.01 (0.88-1.17)	0.92 (0.75-1.13)
	Adequate	1	1	1	1	1	1
	Excessive	1.05 (0.85-1.31)	1.06 (0.88-1.28)	1.00 (0.83-1.20)	0.94 (0.79-1.11)	0.97 (0.84-1.13)	0.84 (0.67-1.04)
9 years	Pre-Pregnancy BMI (*n* = 5683)						
	<18.5	1.69 (1.14-2.48)	1.51 (1.07-2.15)	1.09 (0.75-1.58)	1.50 (1.05-2.15)	1.19 (0.84-1.69)	1.32 (0.85-2.07)
	18.5 – 24.99	1	1	1	1	1	1
	25 – 29.99	1.17 (0.91-1.51)	0.92 (0.73-1.16)	1.14 (0.93-1.40)	1.14 (0.92-1.43)	1.12 (0.92-1.36)	0.83 (0.62-1.12)
	≥30	1.27 (0.86-1.89)	1.38 (0.99-1.92)	1.84 (1.38-2.47)	1.18 (0.83-1.68)	1.35 (0.99-1.83)	0.36 (0.17-0.72)

	Gestational weight gain (*n* = 5443)						
	Inadequate	1.07 (0.86-1.32)	0.97 (0.80-1.17)	1.21 (1.02-1.45)	1.17 (0.97-1.41)	1.03 (0.87-1.22)	0.91 (0.73-1.15)
	Adequate	1	1	1	1	1	1
	Excessive	0.96 (0.76-1.21)	1.03 (0.84-1.25)	1.30 (1.08-1.56)	1.04 (0.85-1.27)	0.98 (0.82-1.17)	0.68 (0.52-0.88)
11 years	Pre-Pregnancy BMI (*n* = 5268)						
	<18.5	1.30 (0.83-2.03)	1.64 (1.14-2.37)	0.95 (0.64-1.42)	1.39 (0.93-.07)	0.99 (0.66-1.47)	0.92 (0.52-1.59)
	18.5 – 24.99	1	1	1	1	1	1
	25 – 29.99	1.27(0.98-1.65)	1.10 (0.86-1.39)	1.15 (0.93-1.42)	1.28 (1.01-1.62)	120 (0.97-1.48)	0.88 (0.64-1.21)
	≥30	1.06 (0.68-1.64)	1.26 (0.88-1.82)	1.35 (0.97-1.87)	0.99 (0.66-1.50)	1.24 (0.88-173)	0.75 (0.43-1.29)
	Gestational weight gain (*n* = 5033)						
	Inadequate	1.05 (0.84-1.33)	0.89 (0.73-1.09)	0.92 (0.77-1.11)	0.98 (0.80-1.22)	0.91 (0.76-1.09)	0.85 (0.66-1.08)
	Adequate	1	1	1	1	1	1
	Excessive	1.13 (0.89-1.44)	1.08 (0.87-1.32)	1.09 (0.90-1.31)	1.08 (0.87-1.33)	0.89 (0.74-1.09)	0.65 (0.49-0.86)
16 years	Pre-Pregnancy BMI (*n* = 4098)						
	<18.5	1.42 (0.85-2.39)	1.88 (1.28-2.76)	1.27 (0.83-1.93)	2.07 (1.35-3.16)	1.58 (1.04-2.41)	1.52 (0.99-2.33)
	18.5 – 24.99	1	1	1	1	1	1
	25 – 29.99	1.08 (0.78-1.50)	0.92 (0.70-1.21)	1.28 (1.01-1.63)	0.96 (0.71-1.32)	1.10 (0.84-1.44)	0.83 (0.63-1.13)
	≥30	1.26 (0.75-2.11)	1.30 (0.87-1.95)	1.33 (0.90-1.97)	0.94 (0.56-1.59)	1.36 (0.90-2.06)	0.63 (0.36-1.09)
	Gestational weight gain (*n* = 3925)						
	Inadequate	0.92 (0.70-1.22)	1.13 (0.91-1.40)	0.95 (0.77-1.17)	1.19 (0.93-1.53)	1.05 (0.84-1.32)	1.06 (0.84-1.33)
	Adequate	1	1	1	1	1	1
	Excessive	1.08 (0.81-1.43)	1.06 (0.84-1.33)	1.15 (0.93-1.43)	1.14 (0.87-1.49)	1.10 (0.87-1.40)	1.09 (0.85-1.39)

Offspring age	Pre-pregnancy BMI and gestational weight gain	**[B] Adjusted; OR (95%CI)#**					
		Total behavioural difficulties	Emotional symptoms	Peer-relationship problems	Hyperactivity/ inattention problems	Conduct problems	Prosocial behaviours
3 years	Pre-Pregnancy BMI (*n* = 7142)						
	<18.5	1.18 (0.93-1.50)	1.22 (0.94-1.58)	…	0.83 (0.52-1.34)	1.16 (0.91-1.50)	…
	18.5 – 24.99	1	1	…	1	1	…
	25 – 29.99	1.06 (0.93-1.22)	1.08 (0.93-1.27)	…	1.13 (0.88-1.46)	1.21 (1.05-1.39)	…

	≥30	1.00 (0.80-1.24)	1.02 (0.79-1.30)	…	1.03 (0.67-1.56)	1.00 (0.79-1.25)	…
	Gestational weight gain (*n* = 6822)						
	Inadequate	1.07 (0.95-1.20)	1.04 (0.92-1.19)	…	1.1 (0.88-1.37)	1.00 (0.89-1.13)	…
	Adequate	1	1	…	1	1	…
	Excessive	0.94 (0.83-1.06)	1.09 (0.95-1.24)	…	1.00 (0.79-1.26)	0.89 (0.78-1.00)	…
7 years	Pre-Pregnancy BMI (*n* = 6159)						
	<18.5	1.38 (0.94-2.01)	1.65 (1.20-2.28)	1.33 (0.95-1.87)	1.19 (0.86-1.65)	0.98 (0.72-1.32)	1.25 (0.82-1.88)
	18.5 – 24.99	1	1	1	1	1	1
	25 – 29.99	1.11 (0.87-1.41)	0.92 (0.74-1.15)	1.17 (0.95-1.44)	1.08 (0.89-1.31)	1.04 (0.87-1.23)	1.11 (0.87-1.43)
	≥30	1.12 (0.77-1.62)	0.92 (0.64-1.31)	1.30 (0.94-1.79)	1.13 (0.83-1.53)	1.18 (0.90-1.54)	0.83 (0.53-1.29)
	Gestational weight gain (*n* = 5912)						
	Inadequate	1.14 (0.92-1.40)	0.98 (0.82-1.18)	0.90 (0.75-1.08)	1.04 (0.88-1.22)	0.99 (0.85-1.14)	0.89 (0.73-1.10)
	Adequate	1	1	1	1	1	1
	Excessive	0.96 (0.77-1.20)	0.99 (0.82-1.20)	0.95 (0.79-1.15)	0.89 (0.75-1.06)	0.94 (0.80-1.09)	0.90 (0.72-1.13)
9 years	Pre-Pregnancy BMI (*n* = 5683)						
	<18.5	1.50 (1.00-2.23)	1.35 (0.94-1.94)	1.01 (0.70-1.48)	1.35 (0.93-1.95)	1.02 (0.72-1.45)	1.41 (0.90-2.22)
	18.5 – 24.99	1	1	1	1	1	1
	25 – 29.99	1.13 (0.88-1.47)	0.89 (0.71-1.14)	1.11 (0.90-1.38)	1.10 (0.87-1.38)	1.11 (0.90-1.36)	0.90 (0.85-1.20)
	≥30	1.15 (0.77-1.72)	1.27 (0.90-1.79)	1.78 (1.31-2.41)	1.09 (0.75-1.57)	1.29 (0.94-1.77)	0.38 (0.18-0.77)
	Gestational weight gain (*n* = 5443)						
	Inadequate	1.06 (0.85-1.32)	0.97 (0.80-1.17)	1.24 (1.03-1.49)	1.17 (0.97-1.42)	1.01 (0.85-1.20)	0.90 (0.71-1.14)
	Adequate	1	1	1	1	1	1
	Excessive	0.89 (0.70-1.12)	0.98 (0.80-1.19)	1.22 (1.01-1.47)	0.97 (0.78-1.19)	0.92 (0.77-1.11)	0.71 (0.54-0.92)
11 years	Pre-Pregnancy BMI (*n* = 5268)						
	<18.5	1.09 (0.69-1.73)	1.49 (1.02-2.18)	0.88 (0.58-1.32)	1.27 (0.84-1.92)	0.82 (0.55-1.24)	0.92 (0.52-1.61)
	18.5 – 24.99	1	1	1	1	1	1
	25 – 29.99	1.19 (0.91-1.55)	1.02 (0.80-1.31)	1.13 (0.90-1.40)	1.24 (0.97-1.59)	1.19 (0.95-1.48)	0.91 (0.66-1.26)
	≥30	0.86 (0.55-1.37)	1.07 (0.73-1.56)	1.26 (0.90-1.76)	0.91 (0.59-1.39)	1.20 (0.85-1.71)	0.74 (0.42-1.31)
	Gestational weight gain (*n* = 5033)						
	Inadequate	1.00 (0.79-1.27)	0.87 (0.71-1.07)	0.92 (0.77-1.11)	0.94 (0.76-1.16)	0.87 (0.73-1.05)	0.83 (0.65-1.07)
	Adequate	1	1	1	1	1	1
	Excessive	1.02 (0.79-1.30)	0.99 (0.80-1.22)	1.02 (0.84-1.24)	1.02 (0.82-1.28)	0.83 (0.68-1.01)	0.63 (0.47-0.84)

16 years	Pre-Pregnancy BMI (*n* = 4098)						
	<18.5	1.22 (0.72-2.09)	1.74 (1.16-2.60)	1.27 (0.83-1.94)	1.96 (1.27-3.05)	1.46 (0.95-2.24)	1.52 (0.98-2.34)
	18.5 – 24.99	1	1	1	1	1	1
	25 – 29.99	1.01 (0.73-1.42)	0.88 (0.67-1.17)	1.22 (0.95-1.56)	0.92 (0.67-1.27)	1.07 (0.81-1.41)	0.84 (0.62-1.14)
	≥30	1.07 (0.63-1.82)	1.14 (0.74-1.74)	1.17 (0.78-1.75)	0.84 (0.48-1.44)	1.23 (0.80-1.89)	0.61 (0.35-1.07)
	Gestational weight gain (*n* = 3925)						
	Inadequate	0.92 (0.69-1.21)	1.13 (0.91-1.40)	1.00 (0.81-1.24)	1.13 (0.88-1.46)	1.04 (0.83-1.31)	1.06 (0.84-1.34)
	Adequate	1	1	1	1	1	1
	Excessive	0.95 (0.71-1.27)	0.97 (0.77-1.23)	1.06 (0.85-1.33)	1.06 (0.80-1.39)	1.02 (0.80-1.30)	1.05 (0.82-1.34)

··· Not measured/no data available.

Adjusted for maternal age, education, marital status, parity, maternal smoking, alcohol use, anxiety and depressive symptoms during pregnancy and offspring sex.

Table S6. Association between pre-pregnancy BMI and behavioural problems in children at each time point: using imputed dataset (*n* = 13442).

Offspring age	Pre-pregnancy BMI and gestational weight gain	Adjusted; OR (95%CI)#					
		Total behavioural difficulties	Emotional symptoms	Peer-relationship problems	Hyperactivity/ inattention problems	Conduct problems	Prosocial behaviours
3 years	Pre-Pregnancy BMI (Kg/m^2)						
	<18.5	1.07 (0.88-1.30)	1.28 (0.96-1.45)	…	0.91 (0.61-1.34)	1.03 (0.84-1.26)	…
	18.5 – 24.99	1	1	…	1	1	…
	25 – 29.99	1.01 (0.89-1.15)	1.04 (0.91-1.19)	…	1.09 (0.88-1.36)	1.17 (1.03-1.33)	…
	≥30	0.98 (0.81-1.19)	1.06 (0.85-1.32)	…	1.07 (0.74-1.54)	1.03 (0.85-1.25)	…
	Gestational weight gain			…			…
	Inadequate	1.05 (0.95-1.16)	1.06 (0.94-1.19)	…	1.07 (0.89-1.30)	0.99 (0.89-1.09)	…
	Adequate	1	1	…	1	1	…
	Excessive	0.95 (0.85-1.07)	1.08 (0.96-1.22)	…	0.99(0.81-1.22)	0.91 (0.82-1.03)	…
7 years	Pre-Pregnancy BMI (Kg/m^2)						
	<18.5	1.09 (0.82-1.46)	1.39 (1.08-1.79)	1.09 (0.83-1.41)	1.02 (0.80-1.30)	0.93 (0.74-1.16)	1.13 (0.82-1.54)
	18.5 – 24.99	1	1	1	1	1	1
	25 – 29.99	1.13 (0.93-1.38)	0.96 (0.78-1.17)	1.11 (0.94-1.31)	1.13 (0.96-1.32)	1.07 (0.93-1.23)	1.02 (0.83-1.27)
	≥30	1.18 (0.86-1.61)	0.86 (0.62-1.19)	1.16 (0.88-1.53)	1.11 (0.87-1.41)	1.18 (0.94-1.50)	0.83 (0.58-1.18)
	Gestational weight gain						
	Inadequate	1.03 (0.86-1.24)	0.97 (0.83-1.14)	0.93 (0.78-1.11)	1.03 (0.91-1.17)	0.98 (0.86-1.11)	0.92 (0.78-1.10)
	Adequate	1	1	1	1	1	1
	Excessive	0.97 (0.79-1.17)	1.01 (0.86-1.19)	0.99 (0.84-1.16)	0.95 (0.82-1.11)	0.93 (0.80-1.07)	0.92 (0.75-1.13)
9 years	Pre-Pregnancy BMI (Kg/m^2)						
	<18.5	1.18 (0.88-1.58)	1.19 (0.90-1.57)	1.00 (0.77-1.31)	1.12 (0.85-1.47)	0.97 (0.75-1.25)	1.36 (0.94-1.95)
	18.5 – 24.99	1	1	1	1	1	1
	25 – 29.99	1.08 (0.86-1.35)	0.93 (0.76-1.14)	1.14 (0.97-1.34)	1.09 (0.89-1.33)	1.08 (0.91-1.30)	0.87 (0.67-1.13)
	≥30	1.24 (0.91-1.69)	1.22 (0.89-1.66)	1.53 (1.18-1.99)	1.08 (0.78-1.50)	1.30 (1.00-1.70)	0.49(0.28-0.85)
	Gestational weight gain						
	Inadequate	1.01 (0.84-1.21)	0.97 (0.83-1.14)	1.12 (0.98-1.29)	1.12 (0.96-1.31)	1.00 (0.86-1.16)	0.90 (0.74-1.09)
	Adequate	1	1	1	1	1	1

	Excessive	0.93 (0.76-1.15)	0.98 (0.84-1.15)	1.16 (0.99-1.37)	1.02 (0.85-1.23)	0.95 (0.81-1.11)	0.71 (0.55-0.91)
11 years	Pre-Pregnancy BMI (Kg/m^2)						
	<18.5	1.10 (0.81-1.49)	1.22 (0.92-1.62)	1.04 (0.78-1.38)	1.10 (0.83-1.47)	0.89 (0.68-1.18)	1.10 (0.73-1.68)
	18.5 – 24.99	1	1	1	1	1	1
	25 – 29.99	1.10 (0.89-1.36)	1.00 (0.81-1.22)	1.11 (0.93-1.32)	1.13 (0.93-1.36)	1.15 (0.97-1.35)	0.95 (0.74-1.24)
	≥30	0.99 (0.70-1.34)	1.05 (0.77-1.44)	1.26 (0.96-1.63)	0.99 (0.70-1.40)	1.22 (0.92-1.61)	0.77 (0.51-1.17)
	Gestational weight gain						
	Inadequate	1.05 (0.87-1.27)	1.01 (0.87-1.19)	1.02 (0.87-1.19)	1.04 (0.89-1.23)	094 (0.80-1.09)	0.81 (0.65-1.01)
	Adequate	1	1	1	1	1	1
	Excessive	1.06 (0.87-1.29)	1.07 (0.90-1.27)	1.06 (0.90-1.26)	1.06 (0.87-1.31)	0.86 (0.72-1.03)	0.65 (0.50-0.84)
16 years	Pre-Pregnancy BMI (Kg/m^2)						
	<18.5	1.00 (0.70-1.43)	1.21 (0.88-1.64)	0.97 (0.73-1.34)	1.27 (0.93-1.75)	1.05 (0.77-1.42)	1.35 (0.99-1.84)
	18.5 – 24.99	1	1	1	1	1	1
	25 – 29.99	1.04 (0.82-1.30)	0.96 (0.76-1.21)	1.24 (1.03-1.48)	0.92 (0.73-1.16)	1.00 (0.82-1.23)	0.82 (0.64-1.03)
	≥30	1.13 (0.78-1.64)	1.09 (0.77-1.54)	1.51 (1.10-2.05)	0.80 (0.73-1.16)	1.10 (0.77-1.58)	0.61 (0.40-0.95)
	Gestational weight gain						
	Inadequate	0.95 (0.77-1.17	1.03 (0.66-1.24)	1.02 (0.87-1.20)	1.05 (0.86-1.28)	1.00 (0.84-1.19)	0.99 (0.83-1.17)
	Adequate	1	1	1	1	1	1
	Excessive	0.89 (0.70-1.12)	0.97 (0.78-1.20)	1.07 (0.88-1.30)	0.98 (0.77-1.26)	0.92 (0.75-1.13)	0.92 (0.72-1.16)

··· Not measured/no data available.

Adjusted for maternal age, education, marital status, parity, maternal smoking and alcohol use during pregnancy, antenatal anxiety and depressive symptoms, and offspring sex.

Table S7. Association between paternal BMI and behavioural problems in children before and after adjustment for maternal BMI (GEE model).

**Paternal BMI (Kg/m²)**	**Crude OR (95%CI)#**					
	Total behavioural difficulties	Hyperactivity/ inattention problems	Conduct problems	Emotional symptoms	Peer-relationship problems	Pro-social behaviours
<18.5	0.60 (0.30-1.23)	0.67 (0.26-1.77)	0.77 (0.49-1.23)	1.10 (0.59-2.05)	0.84 (0.36-1.95)	0.67 (0.23-1.99)
18.5 – 24.99	1	1	1	1	1	1
25 – 29.99	1.01 (0.92-1.10)	1.00 (0.89-1.14)	0.97 (0.91-1.04)	0.92 (0.83-1.01)	1.02 (0.90-1.15)	0.84 (0.73-0.98)
≥30	1.10 (0.93-1.29)	1.40 (1.13-1.73)	1.03 (0.91-1.17)	0.94 (0.78-1.13)	1.37 (1.11-1.69)	1.11 (0.86-1.44)
Paternal BMI (Kg/m²)	**Adjusted OR (95%CI)#**					
<18.5	0.62 (0.31-1.24)	0.74 (0.29-1.93)	0.79 (0.50-1.25)	1.08 (0.59-2.00)	0.85 (0.37-1.97)	0.73 (0.25-2.13)
18.5 – 24.99	1	1	1	1	1	1
25 – 29.99	1.01 (0.92-1.10)	1.00 (0.88-1.13)	0.97 (0.91-1.04)	0.91 (0.83-1.00)	1.01 (0.89-1.13)	0.84 (0.72-0.97)
≥30	1.07 (0.91-1.26)	1.35 (1.09-1.67)	1.02 (0.90-1.16)	0.92 (0.76-1.11)	1.36 (1.10-1.68)	1.13 (0.88-1.47)
Paternal BMI (Kg/m²)	**Adjusted OR (95%CI)¥**					
<18.5	0.62 (0.31-1.25)	0.76 (0.29-1.97)	0.79 (0.50-1.26)	1.07 (0.58-1.98)	0.81 (0.35-1.88)	0.75 (0.26-2.22)
18.5 – 24.99	1	1	1	1	1	1
25 – 29.99	1.03 (0.94-1.13)	1.00 (0.88-1.14)	0.98 (0.91-1.05)	0.91 (0.83-1.01)	0.99 (0.88-1.12)	0.85 (0.73-0.98)
≥30	1.08 (0.91-1.29)	1.39 (1.11-1.74)	1.01 (0.88-1.15)	0.89 (0.73-1.09)	1.29 (1.03-1.61)	1.15 (0.87-1.51)

Adjusted for maternal age, education, marital status, parity, maternal smoking, alcohol use, anxiety and depressive symptoms during pregnancy, and offspring sex .

¥ Additionally adjusted for maternal pre-pregnancy BMI.

Table S8. Maternal BMI and offspring behavioural problems over time: accounted for paternal BMI.

Pre-pregnancy BMI (Kg/m[2])	AOR (95%CI)#					
	Total behavioural difficulties	Hyperactivity/ inattention problems	Conduct problems	Emotional symptoms	Peer-relationship problems	Pro-social behaviours
<18.5	1.14 (0.93-1.40)	1.11 (0.83-1.47)	1.00 (0.85-1.17)	1.35 (1.09-1.69)	1.35 (1.03-1.76)	1.37 (1.01-1.87)
18.5 – 24.99	1	1	1	1	1	1
25 – 29.99	1.09 (0.97-1.23)	1.04 (0.88-1.23)	1.08 (0.98-1.18)	1.06 (0.92-1.21)	1.18 (1.00-1.38)	0.87 (0.71-1.07)
≥30	0.99 (0.81-1.21)	0.77 (0.57-1.04)	1.02 (0.88-1.19)	1.17 (0.94-1.45)	1.42 (1.10-1.82)	0.58 (0.39-0.88)

Adjusted for maternal age, education, marital status, parity, maternal smoking, alcohol use, anxiety and depressive symptoms during pregnancy, offspring sex and paternal BMI.

Table S9. Paternal BMI and offspring behavioural problems at each time points.

Offspring age	Pre-pregnancy BMI (Kg/m[2])	AOR (95%CI) #					
		Total behavioural difficulties	Emotional symptoms	Peer-relationship problems	Hyperactivity/ inattention problems	Conduct problems	Prosocial behaviours
3 years	Pre-Pregnancy BMI (n = 5188)						
	<18.5	0.52 (0.20-1.30)	0.65 (0.24-1.76)	…	…	0.85 (0.38-1.87)	…
	18.5 – 24.99	1	1	…	1	1	…
	25 – 29.99	1.02 (0.91-1.56)	0.96 (0.84-1.10)	…	0.95 (0.76-1.19)	0.94 (0.84-1.06)	…
	≥30	1.05 (0.84-1.33)	0.97 (0.75-1.26)	…	1.31 (0.88-1.94)	0.98 (0.78-1.24)	…
7 years	Pre-Pregnancy BMI (n = 4547)						
	<18.5	1.67 (0.48-576)	1.33 (0.45-3.94)	0.86 (0.25-2.93)	1.14 (0.38-3.43)	1.19 (0.47-3.04)	2.02 (0.66-6.15)
	18.5 – 24.99	1	1	1	1	1	1
	25 – 29.99	1.07 (0.86-1.33)	0.89 (0.74-1.08)	0.96 (0.80-1.15)	1.14 (0.96-1.34)	0.96 (0.83-1.12)	0.85 (068-1.05)
	≥30	1.33 (0.92-1.95)	0.78 (0.54-1.14)	1.09 (0.78-1.53)	1.38 (1.02-1.87)	1.07 (0.81-1.42)	1.23 (0.84-1.80)
9 years	Pre-Pregnancy BMI (n = 4263)						

	<18.5	0.54 (0.07-4.07)	0.99 (0.29-3.41)	0.63 (0.15-2.77)	0.79 (0.18-3.50)	1.22 0.40-3.68)	…
	18.5 – 24.99	1	1	1	1	1	1
	25 – 29.99	1.09 (0.87-1.36)	0.84 (0.89-1.02)	1.06 (0.88-1.27)	1.02 (0.83-1.24)	1.01 (0.84-120)	0.74 (0.57-0.95)
	≥30	1.27 (0.84-1.90)	0.94 (0.64-1.36)	1.43 (1.03-1.98)	1.52 (1.07-2.14)	0.89 (0.63-1.27)	1.17 (0.74-1.84)
11 years	Pre-Pregnancy BMI (n = 3931)						
	<18.5	…	126 (0.36-4.42)	0.59 (0.14-2.59)	1.59 (0.45-5.66)	0.66 (0.15-2.86)	…
	18.5 – 24.99	1	1	1	1	1	1
	25 – 29.99	0.98 (0.771.25)	0.91 (0.74-1.13)	1.00 (0.82-1.21)	0.92 (0.74-1.15)	0.97 (0.80-1.17)	0.6 (0.74-1.25)
	≥30	1.09 (0.69-1.72)	0.88 (0.58-1.34)	1.58 (1.13-2.21)	1.38 (0.93-2.04)	0.97 (0.67-1.41)	1.28 (0.78-2.09)
16 years	Pre-Pregnancy BMI (n = 3160)						
	<18.5	1.25 (0.27-5.68)	2.74 (0.97-7.78)	1.18 (0.38-3.67)	0.57 (0.07-4.41)	0.36 (0.04-2.78)	0.90 (0.20-4.0)
	18.5 – 24.99	1	1	1	1	1	1
	25 – 29.99	1.01 (0.77-1.34)	0.84 (0.67-1.05)	0.86 (0.70-1.07)	0.86 (0.66-112)	0.98 (0.78-1.24)	0.85 (0.67-1.07)
	≥30	0.80 (0.44-1.8)	0.76 (0.47-1.22)	1.15 (0.78-1.71)	1.36 (0.85-2.20)	1.08 (0.69-1.71)	0.96 (0.60-1.55)
	⋯ Not measured/no data available.<br># Adjusted for maternal age, education, marital status, parity, maternal pre-pregnancy BMI, maternal smoking, alcohol use, anxiety and depressive symptoms during pregnancy, and offspring sex.						

Table S10. Association between pre-pregnancy BMI and behavioural problems in children and adolescents over time (GEE model)¥.

Pre-pregnancy BMI and gestational weight gain	OR (95%CI)#					
	Total behavioural difficulties	Hyperactivity/ inattention problems	Conduct problems	Emotional symptoms	Peer-relationship problems	Pro-social behaviours
Pre-pregnancy BMI						
<18.5	1.20 (1.01-1.41)	1.11 (0.88-1.41)	1.06 (0.92-1.21)	1.28 (1.07-1.54)	1.13 (0.89-1.43)	1.29 (0.99-1.69)
18.5 – 24.99	1	1	1	1	1	1
25 – 29.99	1.09 (0.98-1.21)	1.06 (0.92-1.22)	1.11 (1.03-1.20)	1.00 (0.90-1.13)	1.17 (1.02-1.34)	0.93 (0.78-1.11)
≥30	1.05 (0.89-1.23)	1.00 (0.80-1.25)	1.06 (0.93-1.20)	1.09 (0.92-1.30)	1.41 (1.15-1.73)	0.63 (0.46-0.89)
Gestational weight gain						
Inadequate	1.06 (0.97-1.16)	1.10 (0.98-1.24)	0.99 (0.93-1.06)	1.01 (0.92-1.11)	1.01 (0.89-1.14)	0.92 (0.79-1.06)
Adequate	1	1	1	1	1	1
Excessive	0.96 (0.87-1.05)	0.96 (0.85-1.09)	0.93 (0.86-0.99)	1.03 (0.93-1.14)	1.07 (0.94-1.21)	0.83 (0.71-0.97)

Adjusted for maternal age, education, marital status, parity, maternal smoking, alcohol use,, anxiety and depressive symptoms during pregnancy, and offspring sex.
¥ including children with low birth weight and preterm birth in the analysis.
